# Needle point bipolar ionization: environmental safety and inactivation of airborne bacteria and corona virus

**DOI:** 10.1007/s11356-025-36441-0

**Published:** 2025-04-29

**Authors:** Dila Aydın Aytekin, Nurten Tetik, Ülkü Alver Şahin, Coşkun Ayvaz, Elif Nurtop, Cansel Vatansever, Füsun Can

**Affiliations:** 1 Environmental Engineering Department, Engineering Faculty, Istanbul University-Cerrahpaşa, Istanbul, Türkiye; 2 Food Engineering, Faculty of Chemistry and Metallurgy, Yıldız Technical University, Istanbul, Türkiye; 3 Unité Des Virus Émergents, Aix Marseille University, IRD 190, INSERM U1207, Marseille, France; 4 Koç University İşbank Center for Infectious Diseases, Istanbul, Türkiye; 5 School of Medicine, Department of Medical Microbiology, Koç University, Istanbul, Türkiye

**Keywords:** Needle point bipolar ionization, Antibacterial, COVID-19, Air purification, Indoor air

## Abstract

Although there is an increasing interest after the COVID-19 pandemic, electronic ionization efficiency and impact on indoor air quality are not yet fully understood, and studies are insufficient. Therefore, in this study, the disinfection efficiency for viruses and bacteria and the change of indoor thermal comfort parameters (temperature, humidity, pressure) and air pollutants (CO_2_, NO_2_, VOC, O_3_, CH_2_O, PM_2.5_, Particle Number (PN) from 0.3 to 10 µm particle sizes) by a portable indoor air cleaner using the needle point bipolar ionization (NPBI) method were investigated. The highest antibacterial activity was achieved at hour 3 with a 99.8% reduction for *Bacillus subtilis*, 99.8% for *Staphylococcus aureus*, 98.8% for *Escherichia coli*, and 99.4% for *Staphylococcus albus*, and sustained at hour 4th. The ions had antiviral activity on surfaces with a 94% TCID50 reduction of the HCoV-229E virus after 2 h of NPBI-on. No significant changes were detected in thermal comfort parameters, NO_2_, and VOC during the NPBI-on. Moreover, it was found that O_3_ and CH_2_O were not generated when the NPBI system was operated in the room for 4 h. Consequently, an average particle number removal rate of 60% can be achieved with the NPBI system in much less time than with the natural decay time.

**Supplementary Information:**

The online version contains supplementary material available at 10.1007/s11356-025-36441-0.

## Introduction

After the Coronavirus disease 2019 (COVID-19) pandemic, the disinfection and sterilization of indoor environments have become more essential. Severe acute respiratory syndrome related coronavirus 2 (SARS-CoV-2) spreads through the air and can remain in the form of aerosols for a long time and be transmitted over long distances (Van Doremalen et al. 2020). In addition, the virus was determined in the range of 0.25–1 μm aerosol size. Depending on the ambient temperature and humidity, these aerosols can stay in the air for hours (3–4 h) (Guo et al. 2020) and can be transported up to 4 m (Sultan et al. 2011). As a result, efforts have accelerated to develop a new technology that will both inactivate viruses and reduce other indoor pollutants.

Various indoor air-cleaning devices use the technologies of mechanical filtration, ultraviolet light (UV), electrostatic filtration (ESP), photocatalytic processes (PCO), and cold plasma generators (Szczotko et al. 2022). These can be used for removing or reducing the particulate matter (PM), volatile organic carbons (VOCs), ozone (O_3_), and microorganisms such as viruses and bacteria (González-Martín et al. 2021). The commonly used technology is mechanical filtration due to advantages such as being widely available, relatively low-cost, high-rated efficiency and excellent extraction capabilities for low particle sizes with HEPA filters and no additional emission of by-products. The second technology is the ESP which has a high efficiency (82–94%) depending on the ionizing power and filter types, and low-pressure drop, and low maintenance requirements. UV technology is only effective at high intensity with sufficient contact time and is more effective for the inactivation of microbes on surfaces. The PCO has been preferred to reduce gaseous pollutants (e.g., aldehydes, aromatics, alkanes, olefins, halogenated hydrocarbons) commonly with adsorbent media to improve effectiveness. Due to some disadvantages of these techniques such as the specified operating life of the filter and the microorganism’s accumulation within the structure of the filter for mechanical filtration, the harmful photochemical effect on humans (D’Orazio et al. 2013) and damaged materials (Teska et al. 2020) when directly exposed to UV radiation and the requirement cleaning for ESP, ionization technologies are becoming increasingly popular.

There is not yet a standard test procedure for electronic technologies that have been increasingly used in recent years to improve indoor air quality and disinfection. However, an important concern with electrically powered air cleaning devices is by-products (Formaldehyde: CH_2_O and O_3_). It is stated that it is essential to ensure the principle of being “ozone-free” when using these technologies (ASHRE 2020; Zhang et al. 2011). Although ionization and oxidation methods have many unknowns in practice, technology is rapidly evolving, and more reliable indoor methods are being developed. One of these is the needle point bipolar ionization (NPBI) method.

Although there have been many studies on the effectiveness of the ionization method for removing surface (Meschke et al. 2009) and airborne bacteria (Hyun et al. 2017; Nunayon et al. 2019; Ratliff et al. 2023) and particles (Pushpawela et al. 2017; Wu et al. 2015; Abu-Hammad et al. 2020), a few studies have been conducted by the NPBI method for removing pathogens and the potential of by-product formation in ventilation ducts (Zeng et al. 2021; Licht et al. 2021) and in transport (Baselga et al. 2023). It was found that the disinfection effect in the aircraft was not satisfactory, but no by-products were produced, and the aircraft was not damaged (Licht et al. 2021). Baselga et al. (2023) studied the efficiency of NPBI installed in the air conditioning unit of the Zaragoza Tram and found that the ionization with a filter in the air conditioning system reduced the concentration of colony-forming units (CFU) of bioaerosols by 46% and 69% after 30 and 60 min. But they did not obtain any benefit against microorganisms on the surfaces of trams. As in the use of the bipolar ionization system (Kormos et al. 2024), it is seen that NPBI systems integrated into ducts did not reduce airborne pathogens efficiently. There may be a few reasons why these studies could not achieve an effective result. Since ions are very short-lived, they may work well when sprayed quickly on the target in the air stream. Disinfection applied to the duct system primarily targets the air that flows through it, with limited impact on the surrounding environment. OH^−^ can be effective in microscale environments (Lakey et al. 2021). Ambient humidity is an important factor in the effect of NPBI. As pointed out by the United States Environmental Pollution Agency (EPA), there are not enough studies in the literature on the NPBI method, so more evidence is needed on its effectiveness and the generation of toxic components (EPA 2023).

Ionization systems can introduce by-products into the indoor air as well as alter existing components in the indoor air. The main indoor air quality parameters are carbon dioxide (CO_2_), VOC, nitrous dioxide (NO_2_), and PM. VOCs are mainly caused by building materials in the environment and come from outdoor air (Kozielska et al. 2020; Bari et al. 2015; Huang et al. 2018). NO_2_ is mostly related to traffic and comes from outdoor air (Salonen et al. 2019). Research conducted during the COVID-19 pandemic has identified indoor CO₂ levels as a potential indicator of population density and, consequently, an increased risk of airborne infection. According to Minguillon et al. (2020), CO₂ concentrations exceeding 800 ppm have been associated with a heightened risk of viral transmission in enclosed spaces. Fine particles (PM_2.5_) are predominantly found in indoor environments, and although indoor concentrations vary by location, they can reach levels 2 to 5 times higher than those in the outdoor environment (Şahin et al. 2012; Yurtseven et al. 2012; Onat et al. 2019). Furthermore, Onat et al. (2017) identified a statistically significant correlation between *Staphylococcus aureus* bacteria and PM_2.5_ concentrations in crowded public vehicles. Therefore, the change in these parameters should be considered when using a disinfection device for indoor air.

In this study, an NPBI device designed for viruses and bacteria inactivation was systematically evaluated to determine its efficiency in air disinfection and its potential as a portable indoor air purifier without any filters. The findings of this research aim to contribute to the growing body of knowledge on air purification technologies and provide insights into the applicability of NPBI as a viable solution for improving indoor air quality.

## Methods

### Description of the NPBI devices

In this study, an indoor air purifier device using the NPBI technology was used in the experiments, and the photo of the device can be seen in Figure S1a in the supplementary. This was produced by Başarı Incorporated Company. The device has “needles” as electrodes made from carbon fibers and attached to the flexible circuit. The device has three different stand fan operating speeds which are 2.68 m^3^/min, 3.26 m^3^/min, and 3.88 m^3^/min. NPBI technology is uniquely different from other ionization systems due to it does not use a dielectric and the power output is controlled to less than 12.07 eV to prevent the formation of O_3_. The energy required to ionize oxygen in the air should be above 12.07 eV to produce O_3_ (Waddell 2019; Krull et al. 2020). The water vapor in the air is ionized to hydrogen (H^+^) and hydroxide ions (OH^−^) by NPBI. Ions released from the device remove hydrogen from the pathogen, as positive and negative ions surround air particles containing pathogens (e.g., viruses, bacteria, mold spores). In the case of a virus, hydrogen is pulled from the protein shell or capsid. Hydrogen is an essential component of the true structure of the viral protein coat, and without it the virus cannot be infective. In the case of bacteria, when the hydrogen is removed, the cell ruptures and the pathogen die, thus preventing infection.

Total ions released from the NPBI device with the highest fan speed were measured using the ion measurement device (AlphaLab Air Ion Counter, AIC2M) at different distances and the results are shown in Figure S1b. The highest total ion amount (20.10^6^ ions/cm^3^) was observed at 15 cm airflow distance from the device. When the measurement was made at a 1 m distance, this value decreased to < 1.10^6^ ions/cm^3^, which corresponds to indoor air conditions. Moreover, the measurements presented that the ions assume their highest values in the direction of the airflow and do not exceed 1.10^6^ ions/cm^3^ at the vertical and lateral distances of 15 cm from the device.

### Experimental design

**Fig. 1:**
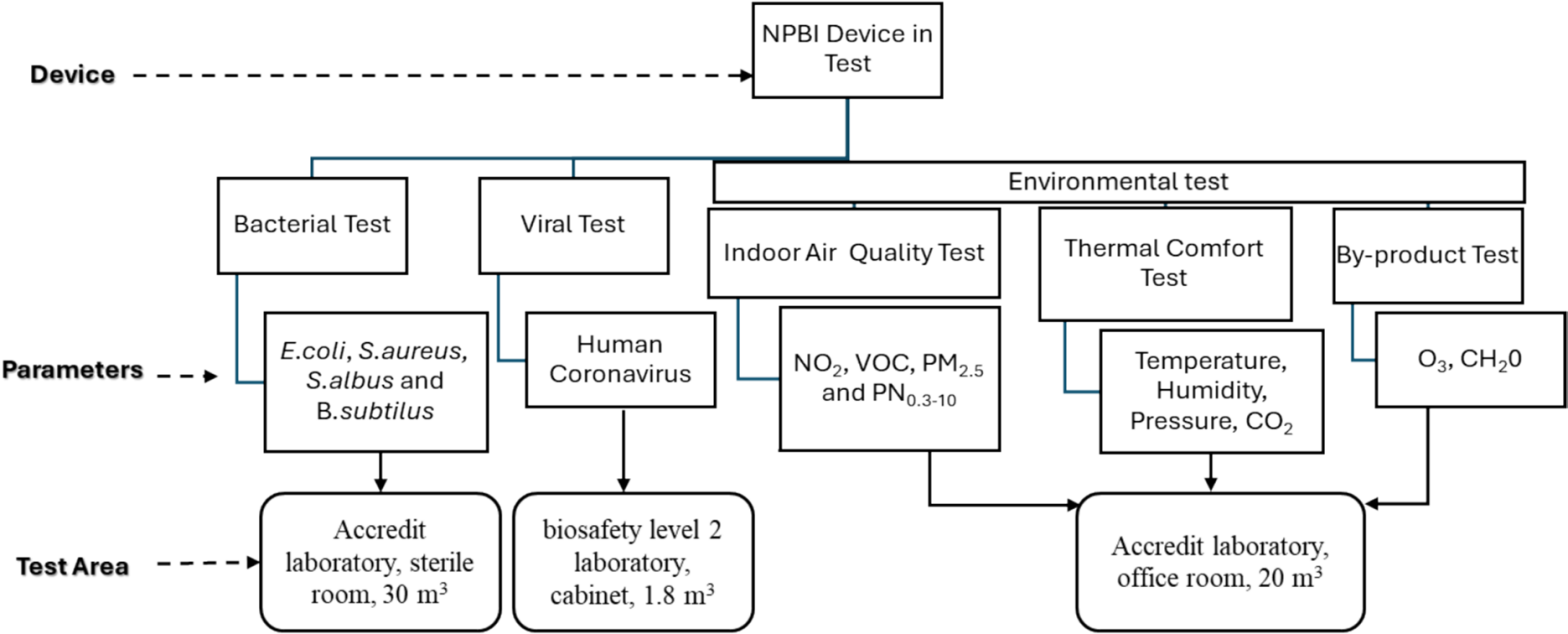
A diagram of the experiment system

For the experimental phase of the study, the effectiveness of a portable air purification system including only the NPBI technology was tested for airborne *Human Coronavirus 229E* and four different bacterial species; *Escherichia coli ATCC* (American Type Culture Collection) 10536 (*E. coli*), *Staphylococcus aureus ATCC* 653 (*S. aureus*), *Staphylococcus albus* 8032 (*S. albus*) and *Bacillus subtilis* ATCC 9372 (*B. subtilus*). In addition, the change in indoor air quality (NO_2_, VOC, PM_2.5_, and particle numbers from 0.3 to 10 µm diameter: PN_0.3–10_) and thermal comfort parameters (temperature, humidity, pressure, CO_2_) were tested. Moreover, it was tested whether the NPBI system can form oxidative by-products (O_3_ and CH_2_O) during continuous long-term operation in a closed indoor environment. This is the first study to test all aspects of the NPBI system for use as a portable air purifier for all parameters together. Each of the tests of bacteria, viruses, and indoor air quality parameters was conducted in different study areas. The tests were performed in nationally accredited laboratories or university laboratories specialized in these analyses. Figure 1 shows the diagram of the experiment system.

### Viral test

Human Corona Virus 229E (HCoV-229E, ATCC Catalog no: VR-740) was cultured MRC-5 cell line (ATCC CCL-171) with Dulbecco minimal essential medium (DMEM) supplemented with 5% fetal bovine serum and antibiotic/antifungal. In the experimental protocol, 2.5 × 10^5^ TCID50/mL HCoV-229E in 100 ml DMEM was used. The viral reduction efficiency of the NPBI device was carried out in a different cabinet than the bacterial study. The device was operated at the highest fan setting. The cabinet was a PVC and plexi-proof cabinet with dimensions of 103 cm, 195 cm, 91 cm. Before the study, the sealing tests of the cabin were checked, and the cabin was in the biosafety level 2 laboratory. Besides, the level of ions in the cabin was measured before starting the experimental protocol.

Two sonic nebulizer devices producing aerosols with a diameter of 0.5–6 µm were placed in the cabin to generate an aerosol at a rate of 10 ml/10 min, and device outlets were arranged to be within 10 cm of the cabin ceiling. The bioaerosol collection system (SKC BIOLITE Biosampling Device, UK) was placed 80 cm above the ground and operated to collect 12.5 L/min of air at 10-min intervals for a total of 30 min with 3 repeats.

The NPBI device was placed on the table in the middle of the cabinet with the ion blower outlet 60 cm above the ground. During the study, the ambient average temperature and humidity were measured as 26 °C and 67%, respectively. Collected air samples at 0, 10, 20, and 30 min were inoculated on MRC5 cells and incubated at 35 °C for 7 days, and the cells were monitored for viability and the cytopathic effect of the virus. On the 7 th day of viral culture, RT-PCR (Reverse transcription polymerase chain reaction) was performed to determine the amount of virus in the samples. Then the percentage of viral and bacterial reduction was calculated.

### Bacterial test

In bioaerosol sampling, it was performed by an impaction-by-impaction method, which is a technique with a high collection rate, in which bioaerosols are collected directly in the culture medium (Grinshpun et al. 2015). Sampling was carried out in a sterile room of 30 m^3^ (3.5 m × 3.4 m × 2.5 m) (Figure S2). Monitoring was done with temperature and humidity sensors to control the air-conditioning of the room. During the study, the ambient average temperature and humidity varied by 20–22 °C and 50–60%, respectively. Bacterial solutions were injected into the room with the nebulizer system at the air flow rate of 28.3 L/min located 1.78 cm above the floor. The air of the test room was cleaned with a HEPA filter (for EN 1822 classification H14 class) placed on the ceiling before and after the experiments. In addition, there was a ceiling and a stand fan to ensure homogeneous distribution of bacteria in the room, and a UV-C lamp and a disinfection nebulizer to ensure post-test sterilization (BS 2018; Lee et al. 2019).

Bacteria used in this study were lyophilized from *B. subtilis* ATCC 9372, *S. aureus* ATCC 653, *E. coli* ATCC 10536, and *S. albus* 8032 from Center of Industrial Culture Collection in China. Freshly prepared bacterial cultures were diluted with Maximum Recovery Diluent (MRD) and 10 ml bacterial suspension was prepared at a concentration of 10^9^ cfu/ml for each bacteria species. It was thoroughly mixed with the help of a vortex mixer and ensured to be homogeneous (ISO 2019; GB 2010).

One day before starting the test, sterile controls of the room were provided. The NPBI device was placed in the middle of the room, 1 m above the floor. The device was operated at the highest fan setting. 6 ml of bacterial suspension was put into the nebulizer system. With an air flow of 28.3 L per minute, the solution was scattered in the chamber air as an aerosol for 10 min. After this process, the initial sample (0. minute/control) is taken from the room with the air sampling device (Diatek Hytest Air). Then, bioaerosol samples were taken at 10., 20., 30., 60., 120., 180., 240. min during the operation of the NPBI device. 1000 L of air were drawn with each sampling device. After sampling, all petri dishes were incubated under appropriate conditions. A room background study was performed to determine the natural decay of bacteria under the same operating conditions. At the end of incubation, colonies were counted, and cfu/ml was calculated.

### Environmental test

The change in indoor air quality that occurs when the NPBI device is operated in an enclosed space was studied. For this purpose, the experiments were conducted under closed conditions in an empty 20 m^3^ office room on the second floor of the building (there is only a small wooden table on which the measuring instruments are placed). The building is located near the arterial road. Separate tests were performed under the condition of three different stand fan operating speeds of the NPBI device (WM1: work mode of 1 st stage: 2.68 m^3^/min; WM2: 2nd stage: 3.26 m^3^/min; WM3: 3rd stage: 3.88 m^3^/min). The parameters monitored in indoor air are the PM_2.5_, NO_2_, VOC, CO_2_, CH_2_O, O_3_ humidity, temperature, pressure, and particle number (PN) of 0.3, 0.5, 1, 3, 5, 10 µm sizes. Thermal comfort and air quality parameters were measured by a NEMo XT Indoor air quality monitor (ETHERA, France) which is an online air quality analysis station and has all air quality parameters sensors selected in this study. Designed to be permanently wall-mounted, it is electrically powered. Compatible with IoT or wired networks, it is easy to install in any type of building. The measurement range and accuracy (in brackets) of the parameters measured during the experiment are 0–280 ppb (down to 1 ppb) for CH_2_O, 0–5000 ppm (± 50 ppb) for CO_2_, 30 ppb-5 ppm (± 40 ppb) for VOC, 0–1000 µg/m^3^ (± 10 µg/m^3^) for PM_2.5_, 1 ppb-17 ppm (± 15 ppb) for NO_2_, 1 ppb-7.6 ppm (± 15 ppb) for O_3_. CO_2_ and VOC are measured with non-dispersive infrared spectrometry (NDIR) and photoionization (PID) sensors, respectively; NO_2_ and O_3_ are measured with electrochemical sensors; CH_2_O is measured with the optical reading of nanoporous material sensor and PM_2.5_ is measured with laser-based light scattering method sensor.

In addition, the O_3_ change was also tested using the reference measurement method, ASTM D 4490–96 (Standard Practice for Measuring the Concentration of Toxic Gases or Vapours Using Detector Tubes). This is an active sampling method, and the pump and sampling O_3_ tube no are Kitagawa/AP-20 Aspirating Pump and 182U, respectively. By attaching inorganic gas sampling tubes to this pump, samples are taken in short periods (3–5 min) and the concentration is determined from the colour change scale in the tubes. We aimed to use this sampling method to check the O_3_ concentration by the standard method. The measurement range and detection limit of this method dependent on the O_3_ tube was 0.025–0.05 ppm and 0.01 ppm, respectively. Furthermore, particle number counts were performed using the Lighthouse HandHeld 3016 particulate matter counter. This device counts the particles in 0.3, 0.5, 1, 3, 5, and 10 µm cut point sizes.

The NPBI device was operated for 4 h in a closed environment. The room was naturally ventilated for at least 1 h before each measurement to ensure real indoor conditions. Then, it was kept closed for 1 h and then the background pollution was observed for 1 h without operating the NPBI device. The NPBI device was placed in the centre of the room. In order not to change the air circulation and concentration in the room, the room was neither entered nor left, and all electrical on/off operations were controlled from the side of the room door. The experiments were conducted in three different operating modes of the NPBI device on different days. Each device was used in separate tests to avoid device interference.

### Statistical analysis

All experiments were performed in triplicates with two biological replicates. Environmental tests for the PM_2.5_, NO_2_, VOC, CO_2_, CH_2_O, O_3_ humidity, temperature, pressure was performed once for each three different stand fan operating speeds of the NPBI device, and particle number (PN) measurement was done once in minimum fan operating condition. For repeated trials, statistical analysis was performed with the Mann–Whitney *U* test using GraphPad Prism Software ver.10.0 (California, US). Error bars represented a standard deviation of the data set relative to the mean. The *p* values of the *t*-test between the average values of the device-off (during an hour) and for each 1-h time span during the device-on for environmental tests were calculated.

## Results and discussion

### Viral studies

**Fig. 2:**
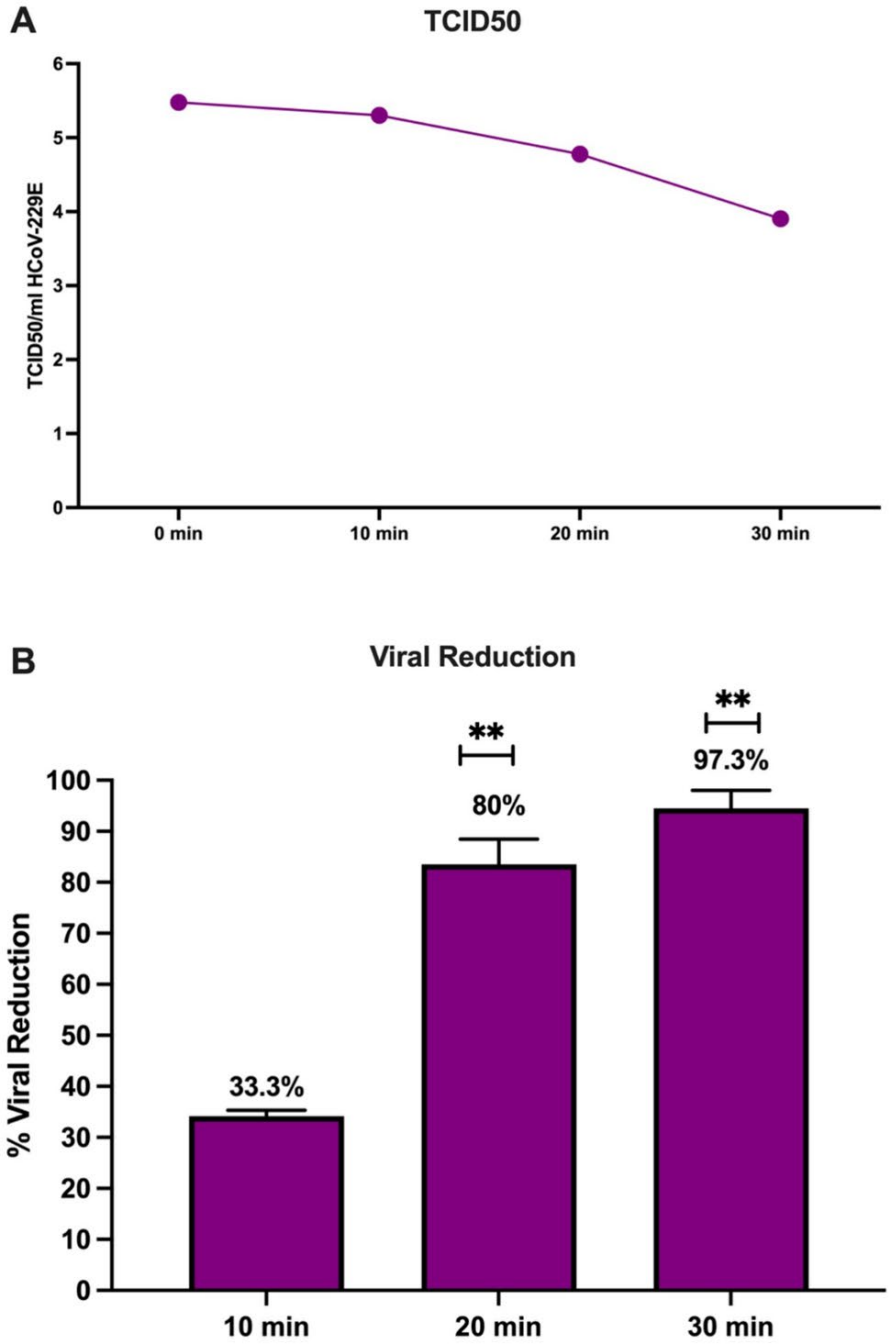
**A**) time-dependent LogTCID50 values of HCoV-229E with bipolar ionization treatment. **B**) Time-dependent % HCoV-229E reduction with bipolar ionization treatment

The TCID50/ml of the virus in the samples collected by operating the device is presented in Fig. 2. We evaluated the effectiveness of the NPBI in reducing the concentration of aerosolized HoCoV 229E in a 1.83 m^3^ sealed cabinet. The experiment was started with HCoV-229E at 3 × 10^5^ TCID50/ml. The bipolar-charged ions inactivated aerosolized HCoV-229E virus at 33.3% (SD = 1.179) in 10 min, 80% (SD = 4.950) in 20 min, and 97.3% (SD = 3.536) in 30 min. After 30 min, TCID50/ml decreased to 8 × 10^3^ (*p* = 0.033). Two recent studies reported similar reduction rates with bipolar ionization as well. In the first study, the positive and negative ions had antiviral activity on surfaces with a 94.0% TCID50 reduction of the HCoV-229E virus after 2 h of exposure (Kanesaka et al. 2022). The second study reported that the bipolar ionization system had reached the maximum antiviral capacity at 60 min of exposure with an approximate 1.1 log10 (91%) reduction in MS2 concentration (Ratliff et al. 2023).

There is a limited number of studies evaluating the antiviral effect of bipolar ionization. The lack of standard guidelines for the assessment of the antiviral effectiveness of this technology is the major limitation in this area. The size of test chambers or air sampling methods is a significant confounding variable that might affect the concentration of ions and viability of viruses in the air. A Japanese team performed a similar experiment in a 3-L chamber and reported a 91.3% reduction in Human Coronavirus 229E concentration in the air (Kanesaka et al. 2022). In another study, the cold plasma bipolar ionization device (PuriFi Labs, Phoenix, AZ) reduced MS2 concentration by 44% at 15 min, 86% at 60 min, and 99.9% at 90 min in a 12 ft × 10 ft × 25 ft (EPA 2021).

### Bacterial studies

**Fig. 3:**
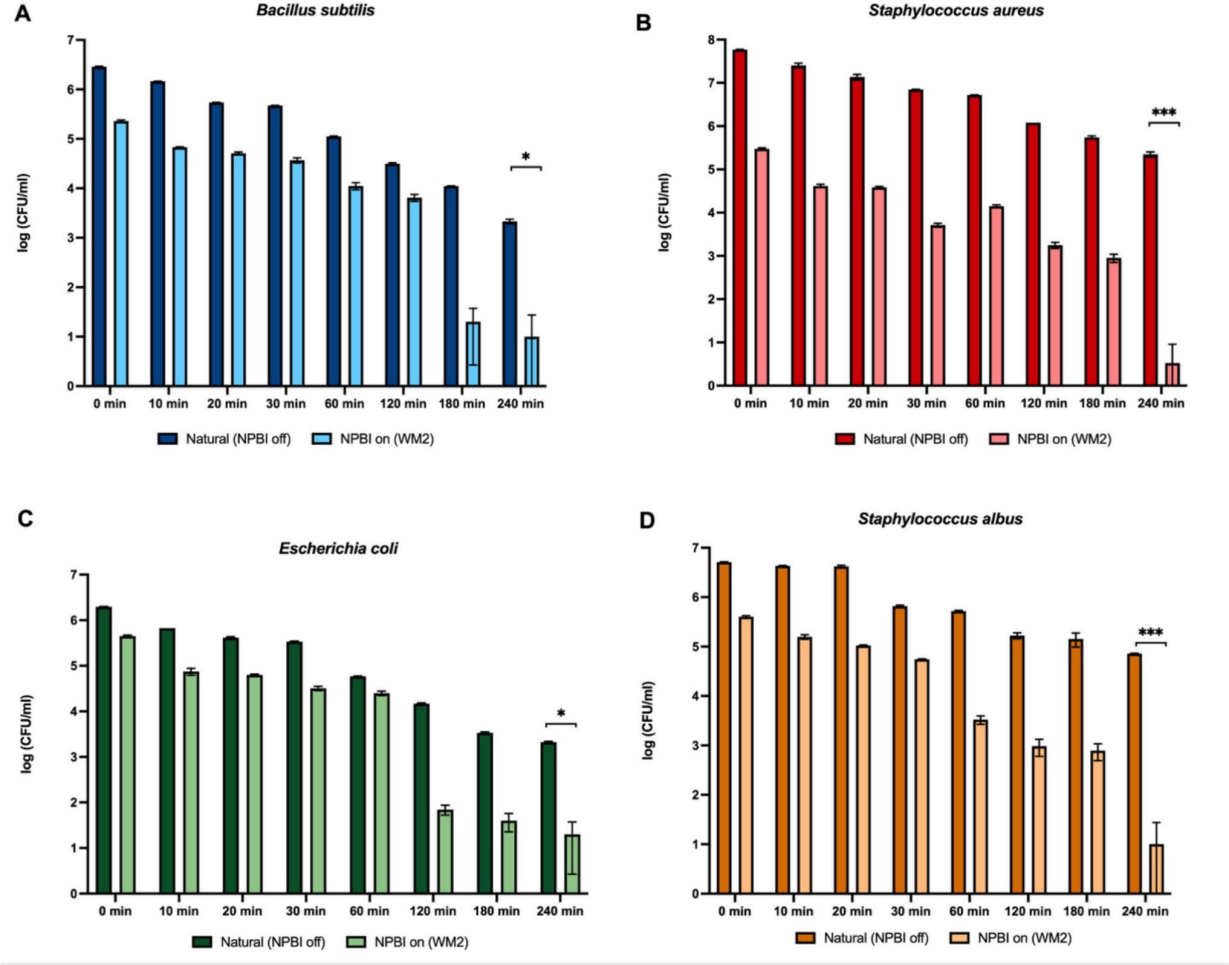
Mean colony counts of different bacterial species within 4 h for natural decay and bacterial recovery during the operation of NPBI device

The total number of bacterial colonies counts in the samples taken during 10.−20.−30.−60.−120.−180.−240. min was calculated for the 30 m^3^ room for the experimental sets with and without the device. Calculated mean values are given in Table S1 and Fig. 3. The decrease in colony numbers over time for the NPBI device is shown in Figure S3.

The significant bacterial inhibition at 4 h after the operation of the NPBI device was detected. The colony counts decreased from 2 × 10^3^ to 10^1^ (2.3 logs; *p* = 0.411) for *B. subtilus*, from 2 × 10^5^ to 1 (4.8 logs; *p* = 0.003) for *S.aureus*, 2 × 10^3^ to 2 × 10^1^ (2 logs; *p* = 0.437) in *E. coli*, 7 × 10^4^ to 10^1^ (3.8 logs; *p* = 0.001) for *S. albus* corresponding > 99% for all bacterial species including spore-forming *B. subtilus*. In the study carried out by Kanesaka et al. (2022), 4 h operation of bipolar ionization showed a 1.23–4.76 log reduction, corresponding to a 94– > 99.9% reduction of pathogenic gram-positive and gram-negative bacteria which were *C. difficile*, *K. pneumoniae*, *Methicillin*-*resistant S. aureus* (MRSA), and *P. aeruginosa*. Despite the efficient disinfection at the 4 h, it is essential to consider the practicality of long-term exposure in real-world applications. There are some other technologies such as non-thermal plasma that can remove bioaerosols containing bacteria and viruses within 90 min in experimental chambers (Li et al. 2024). However, practicality of these technologies in real-world scenarios with dynamic conditions should be further explored.

When we analysed bacterial reduction during the operation of the NPBI device compared to natural decay, we observed time-dependent changes in the activity of the bipolar ionization system. The reduction rates of all bacteria with NPBI systems fluctuated within 60 min (Figure S3-A). After 2 h, the bacterial reduction rate compared to natural decay was 79.3% for *B. subtilis*, 99.8% for *S. aureus*, 99.5% for *E. coli*, and 99.4% for *S. albus*. The highest antibacterial activity was achieved at hour 3 with a 99.8% reduction for *B. subtilis*, 99.8% for a *S. aureus*, 98.8% for *E. coli* and 99.4% for *S. albus*, and sustained at hour 4 th. Likewise, a recent study reported 46%, and 69% of bacterial CFU reduction in 30 and 60 min, respectively (Baselga et al. 2023).

The bacterial inactivation of bipolar ions varies in a range from 20 to 88% against different bacteria species. Gram-negative bacteria are to be more susceptible than Gram-positive bacteria (Lee et al. 2014; Sharp 2020). In our experimental system, the NPBI device showed faster antibacterial activity against Gram-negative *E. coli* and *S. aureus* compared to *S. albus* and *B. subtilus*. *B. subtilus* is a spore-forming bacterium and is known as the most disinfection-resistant pathogen. Similar results were reported in a study which that investigated the anti-bacterial efficiency of bipolar air ions against aerosolized Staphylococcus epidermidis in a 0.04 × 0.04 m^2^ duct flow and reported a maximum 85% bacterial log reduction depending on the exposure time (Nunayon et al. 2022).

### Indoor thermal comfort studies

Considering the real application of the NPBI system, its effect on the indoor parameters of an office was studied during a 4-h performance test. Figure S4 shows the change in indoor parameters, e.g., relative humidity, air temperature, air pressure, and CO_2_ concentration. The air changes per hour (ACH) provided by the unit in rooms for WM1, WM2, and WM3 are approximately 8, 10, and 12, respectively. For infection control in hospitals, it is recommended that the ACH should be between 4 and 6 (Allen and Ibrahim 2021). In the COVID-19 procedure, the use of natural or mechanical ventilation or portable air cleaners with an ACH of 6 and above reduces the risk of transmission (Allen and Ibrahim 2021; Minguillon et al. 2020). For this reason, experiments were conducted in all three WMs and the change in parameters over time was observed. The p values of the *t*-test between the average values of the device-off (during an hour) and for each 1 h during the device-on were given in Tables S2. Table S2 and Figure S4 show that mostly there was no significant difference (*p* > 0.05) between the NPBI device off (shown in dark colour in Figure S4) and on (shown in light colour in Figure S4) during 4 h for indoor air pressure and humidity. On the other hand, it can be observed that the indoor air temperature tends to increase, and p values are below 0.01. Regardless of the operation of the device, an increase in an ambient temperature of 1 °C occurred at the end of the 5-h measurement period in all three operating modes. It is assumed that the main reason for this is that the environment is completely closed, and heat exchange occurs due to the parameter-measuring devices operating in the environment.

The average CO_2_ level of the environment is in the range of 450–500 ppm, which is slightly higher than the CO_2_ level of an outdoor environment (420 ppm). When the NPBI device is put into operation, the CO_2_ level in the indoor air increases slightly due to the person who was in to open the device and then gradually decreases so that at the end of 4 h it is 150–200 ppm. There is mostly a statistically significant difference (*p* < 0.05) in the 1-h averages when the device is switched on and off (Table S2). In the study conducted by (Ye et al. 2021), it was found that negligible CO_2_ is formed by oxidation when air cleaners are used. Another reason for the increase seen in this study is that the very small amounts of carbon monoxide (CO) and hydrocarbons (HC) from traffic are likely to be present and may have converted to CO_2_. Subsequently, a reduction of 5 ppm CO_2_ every 10 min (CO_2_ decay rate = 30 ppm per hour) was found to persist due to the OH^−^ released by the device. The lifetime of OH^−^ in the atmosphere is shorter than one second (Lakey et al. 2021), and it is known that they play a role in reducing the important greenhouse gases such as O_3_, CO_2_, and methane (CH_4_) in the atmosphere (Vimbert et al. 2020; Murray et al. 2021). The CO_2_ reduction in the indoor air when using the NPBI device is something new in the literature and should be supported and explained by strong experiments, analysis, and reaction mechanisms for further studies.

### Indoor air pollutants studies

**Fig. 4:**
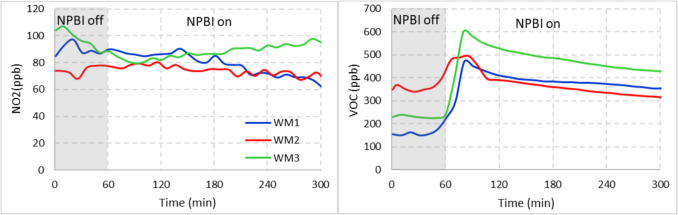
Variation of NO_2_ and VOC pollutants in the office room before and during the operation of NPBI device. WM1: 2.68 m^3^/min; WM2: 3.26 m^3^/min; WM3: 3.88 m^3^/min

The main gaseous pollutant parameters in indoor air pollution are VOC and NO_2_. Figure 4 shows the change in these gas concentrations when the NPBI device is in operation for 4 h. VOCs are mainly caused by building materials in the environment and outdoor air quality (Kozielska et al. 2020; Bari et al. 2015; Huang et al. 2018). In the study room, there is no office material (carpet, chair, furniture, etc.) that could be a source of VOCs, but there are VOCs that are entire because of outdoor air. When the room is empty, the VOCs are in the range of 350–450 ppb. A small decrease was observed in the operation of the NPBI device, and at the end of the 4 th hour there was a VOC reduction of about 100 ppb (~ 20%), especially in the 1 st and 2nd hour (*p* < 0.01, Table S2). When the NPBI device is put into operation, the VOC level in the indoor air increases slightly. It may relate to the person who was in to open the device and may relate to opening the door. Ye et al. (2021) investigated the VOC collection performance of some oxidation and adsorption-based portable air cleaners. They found that the removal of VOC by oxidation is very low, while adsorption is much more effective, and reactive VOC species (such as limonene) are important. The VOC reduction or increase in the indoor air when using the NPBI device, even if small, should be supported and explained by strong experiments and analysis by determining the species of VOCs and reaction mechanisms for further studies. Indoor sources of NO_2_ are mostly related to outdoor air quality (Salonen et al. 2019). In the case of the operation of the device, the formation of NO_2_ is a situation that can only occur due to the oxidation of NO in the environment or the ionization of N_2_. The ionization eV of the NPBI device will not exceed 12 and the N_2_ will not be degraded. As can be seen in Fig. 4, at the end of the 4 th hour, there was a slight decrease in the operating conditions of the 1 st stage compared to the background of the room, but no important change.

**Fig. 5:**
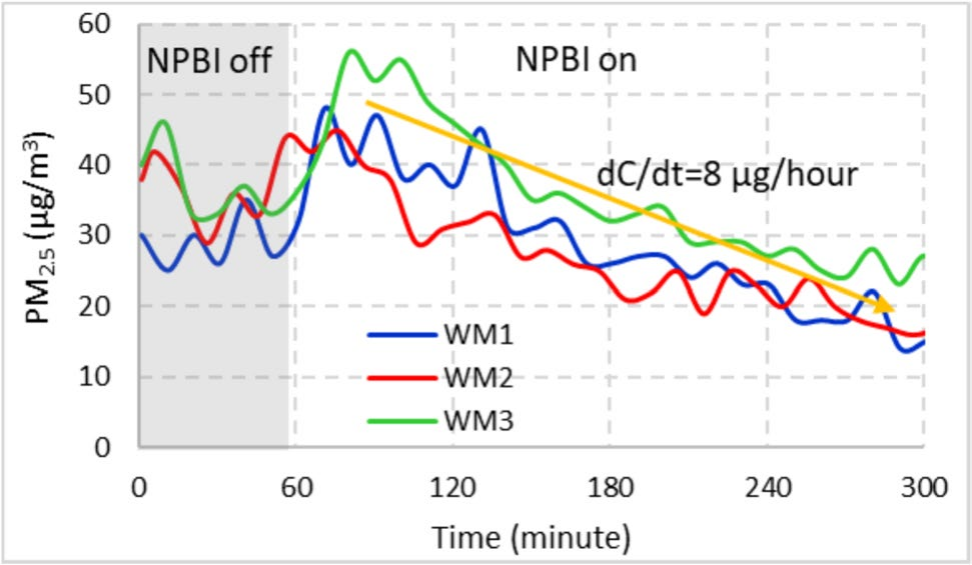
Variation of PM_2.5_ in the office room before and during the operation of NPBI device. WM1: 2.68 m^3^/min; WM2: 3.26 m^3^/min; WM3: 3.88 m^3^/min)

One of the most important parameters in terms of indoor air quality is particulate matter. The change in PM_2.5_ concentration during experiments in office spaces is shown in Fig. 5. The PM_2.5_ concentration in the working environment is 30–40 µg/m^3^ at the beginning and decreases to 15–25 µg/m^3^ at the end of the 4 th hour (~ 60% decrease). A slight increase in PM_2.5_ concentration in the ambient air was observed in the first 30 min after the operation of the NPBI device. Thereafter, there was an average PM_2.5_ reduction of 8 µg/m^3^ per hour (decay rate: dC/dt). Gupta et al. (2023) determined the efficiency of ionizations with filters and tested bipolar air ionizers models showed up to 80% particulate matter (PM_2.5_ and PM_10_) removal efficiencies. In this study, the NPBI system without filter does not show such high reduction efficiency.

**Fig. 6:**
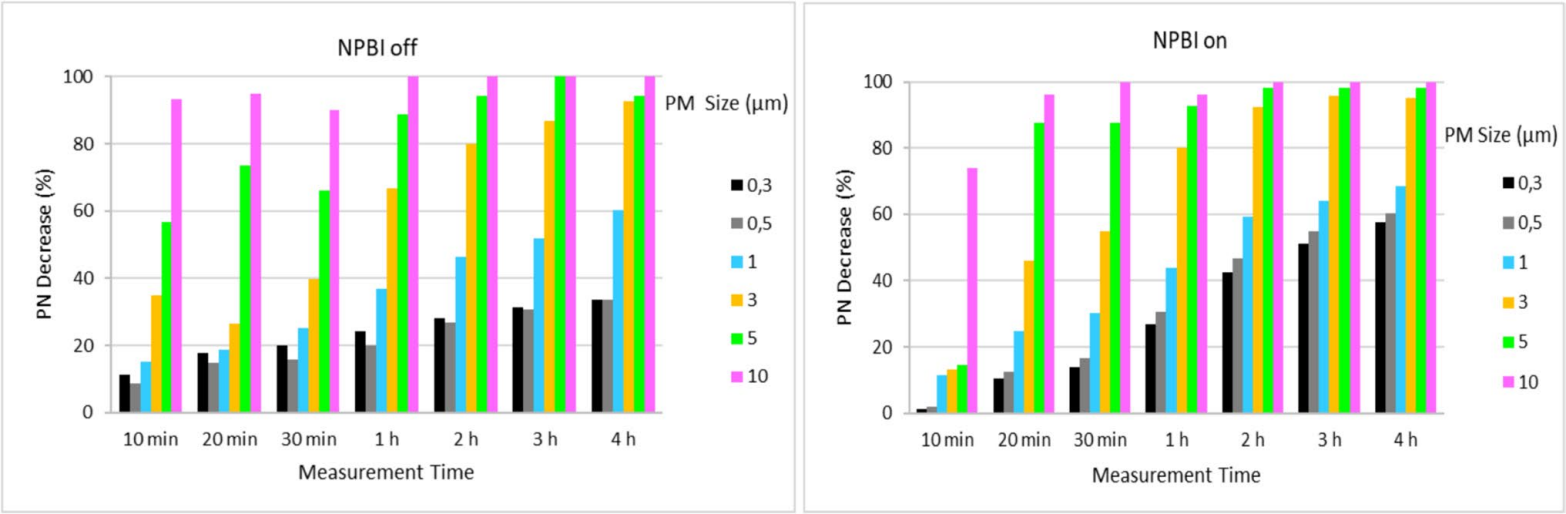
Particle number decrease percentage during the NPBI on and off in the office room

The effect of active operation of the NPBI device during 4 h on the particle counts at 5 different sizes between 0.3 µm and 10 µm was analysed by changing the operating modes of the device. The NPBI did not cause a significant difference in PM_2.5_ removal (Fig. 5) between the working modes. For this reason and since it provides the recommended ACH value, it was investigated in WM1 as a preliminary test for the particle count effects. Figure S5 shows the temporal variation of the numerical concentration of particles in different PM sizes occurring during the operation of the NPBI device and under natural conditions (when the device is turned off). Figure 6 shows the deposition rates of PM at different times for better comparison.

The natural reduction of particulate matter in the first 10 min is higher (94% for 10 µm, 11% for 0.3 µm) compared to the operation of the NPBI device (74% for 10 µm, 11% for 0.3 µm). In the first 5–10 min after switching on the NPBI device, there is a small jump, especially for very fine PMs (< 1 µm), and then a decreasing trend starts. It is considered that this situation arises due to the condensation of gas molecules under the influence of negatively and positively charged ionization in the environment and particle formation or agglomeration processes of electronically charged particles with a size of nm. Particles between 0.1 and 1 µm in the air are particles with accumulation mode, formed from the combination of fine particles by condensation, coagulation, and accumulation processes. Compared to the natural reduction after the first 30 min, the reduction of particle counts for the sizes from 0.3 to 0.5 µm was 1.5–2 times greater (30–60%) by operating the NPBI device. On the other hand, the reduction of 1 µm size particle is 8%, 3 µm size particle is 2.5%, 5 µm size particle is 4% and 10 µm size particle is 0% with NPBI device compared to natural reduction. Consequently, an average removal rate of 60% can be achieved with the NPBI system in much less time than with the natural removal of < 1 µm. According to the review study conducted by Abu-Hammad et al. (2020), the removal rates of aerosols with a diameter of 0.5–2 µm increased by 72% because of corona discharge ionization. Very limited studies have pointed out that the ionization technique is moderate, and O_3_ and UVC techniques are not effective in removing ultra-fine particles. In this study, we could not evaluate since we could not measure below 100 nm. However, our study shows that the effect of ionization technique on the change of particles in the air should be studied with more comprehensive and experimental studies.

### By-product studies

The possibility that the operation of ionization systems may release some gases harmful to human health is the most important factor to consider. The most important of these gases are O_3_ and CH_2_O. According to a study by ASHRAE, indoor O_3_ levels range from 2 to 25 ppb when a device that produces ions using the corona discharge method is turned off, while this level increases to 25–40 ppb when the device is turned on (ASHRE 2020). CH_2_O can be formed because of the reaction of terpenes and other VOC species, depending on indoor conditions, especially in the presence of indoor O_3_. They are formed by the reaction of oxygen radicals, probably released into the environment by ionization of gases, with O_2_ and VOCs. The main advantage of NPBI systems is that they do not form oxygen radicals and do not produce O_3_ and CH_2_O gases. For this purpose, the instantaneous changes in O_3_ and CH_2_O concentration were measured by the continuous monitoring sensor. In addition, the O_3_ presence was also tested using the reference measurement method, ASTM D 4490–96. In all measurements, a value above the measurement limit of 0.01 ppm was not detected. It was found that O_3_ and CH_2_O were not generated even when the NPBI system was actively and continuously operated in the room for 4 h. While there are some studies reported that no by-product formation was observed in indoor air during the ionization device operation (Gupta et al. 2023; Romay et al. 2024), to the best of our knowledge yet, no study for using portable NPBI systems. Baselga et al. (2023) have worked on the effect of NPBI system in the duct of a train, but it was out of the scope of the study. Licht et al. (2021) detected no ozone production within the airplane cabinet using the NPBI system.

## Conclusion

In this study, the NPBI method was investigated, which is a new technology for which there is not yet sufficient evidence. The aim of this study is to demonstrate the use of the NPBI method as a portable indoor air cleaner through a multi-parameter study. The most basic mechanism in ionization systems is the enrichment of molecules in the environment with charge and then the formation of larger particles by the attraction of positive/negative charges and their separation from the environment. In addition, it is expected that the chemical structure of the gas molecules in the environment is changed, and a microbiological inhibition effect occurs. The known electronic ionization methods (ionization, ESP, etc.) can release a significant amount of O_3_ and CH_2_O into the environment, which may pose a risk to human health. To avoid this situation, NPBI systems have been developed that focus on the generation of much shorter-lived OH^−^ instead of oxygen radicals with 12 eV energy. In the experiments carried out with a portable air purifier working continuously with this method for 4 h, the changes of numerous parameters in the indoor air were studied, and the main results are summarized as follows:

- No more than 0.01 ppm O_3_ and CH_2_O were measured in the air,
- The temperature increases by about 1 °C, humidity decreases by about 2%, and there is no significant difference in ambient pressure,
- The CO_2_ level decreases by about 20% compared to the initial value,
- VOC level decreases by about 20% from baseline,
- NO_2_ concentration in the environment does not change,
- PM_2.5_ concentration decreases by about 60% from baseline,
- The number of particles with the size above 2.5 µm does not change significantly compared to the natural reduction,
- After the 30 th minute after the start of the NPBI device, the number of particles with a size of 0.3 to 0.5 µm is reduced by 1.5 to 2 times compared to the natural reduction,
- 94.0% TCID50 reduction of the HCoV-229E virus were detected after 2 h of NPBI device operation,
- The highest antibacterial activity was detected at hour 3 between 99.8% and 99.4%.

This study has some limitations in general. Not all analyses could be performed in the same environment. Analyses could not be tried with more repetitions. The change in parameters with the change in ambient conditions was not considered. In the future, it will be useful to conduct detailed studies that will clarify the following: (i) NPBI systems should be tested at different indoor humidity and temperature values, (ii) the effect of the NPBI method on the size distribution of particulate matter needs to be studied with more experiments to cover a much wider size range of particulate matter; chemical and physical transformations should be described in detail, (iii) the real application range of furniture and goods should be studied and the VOC species/specification change should be investigated, (iv) although the study by Dong et al. (2019) showed that air purifiers using ionization have a positive effect on the respiratory system but have a negative effect on heart rate variability (HRV), there is still no detailed study on the toxic effect of NPBI systems on human health. Multidimensional studies on the toxicological effect should be conducted.

Supplementary information.

## Supplementary Information

Below is the link to the electronic supplementary material.

Supplementary file1 (DOCX 1576 KB)

## Data Availability

We declare that all data relating to this manuscript are truthful and we will gladly share it with any interested readers or at the request of the editor board.
