# Supplementary material for: Needle point bipolar ionization: environmental safety and inactivation of airborne bacteria and corona virus
Source: Environ Sci Pollut Res Int. 2025 Apr 29;32(20):12360–71. doi: 10.1007/s11356-025-36441-0 (PMC12098470; doi:10.1007/s11356-025-36441-0)
Supplement: Supplementary file 1 — Supplementary file1 (DOCX 1576 KB) [file 11356_2025_36441_MOESM1_ESM.docx]

**Needle Point Bipolar Ionization (NPBI): Environmental Safety and Inactivation of Airborne Bacteria and Corona Virus**

^1^Dila Aydın, ^2^Nurten Tetik, ^1^Ülkü Alver Şahin*, ^1^Coşkun Ayvaz, ^3^Elif Nurtop, ^4^Cansel Vatansever, ^4,5^Füsun Can

^1^Istanbul University-Cerrahpaşa, Engineering Faculty, Environmental Engineering Department, Istanbul, Türkiye

^2^Food Engineering, Faculty of Chemistry and Metallurgy, Yıldız Technical University, Istanbul, Türkiye

^3^ Unité des Virus Émergents, Aix Marseille University, IRD 190, INSERM U1207, Marseille, France

^4^ Koç University İşbank Center for Infectious Diseases, Istanbul, Türkiye

^5^Koç University, School of Medicine, Department of Medical Microbiology, Istanbul, Türkiye

*ulkualver@iuc.edu.tr

a)
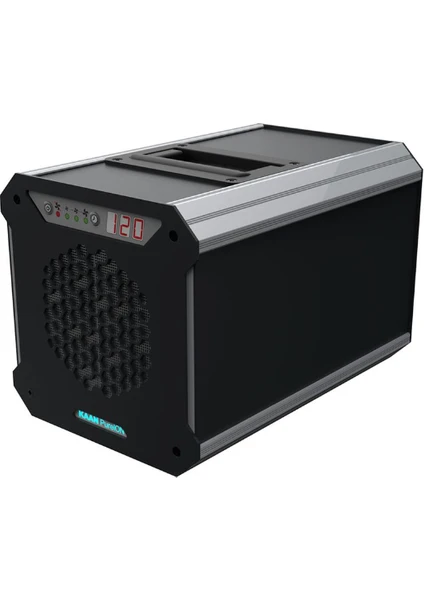
b)
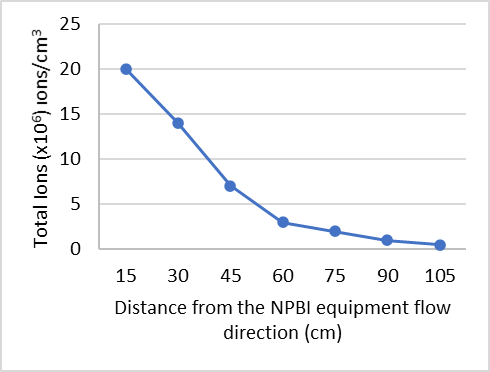


**Figure S1.** Total ions released (a) at different distance from the NPBI device (b) with WM3 operation condition.

**
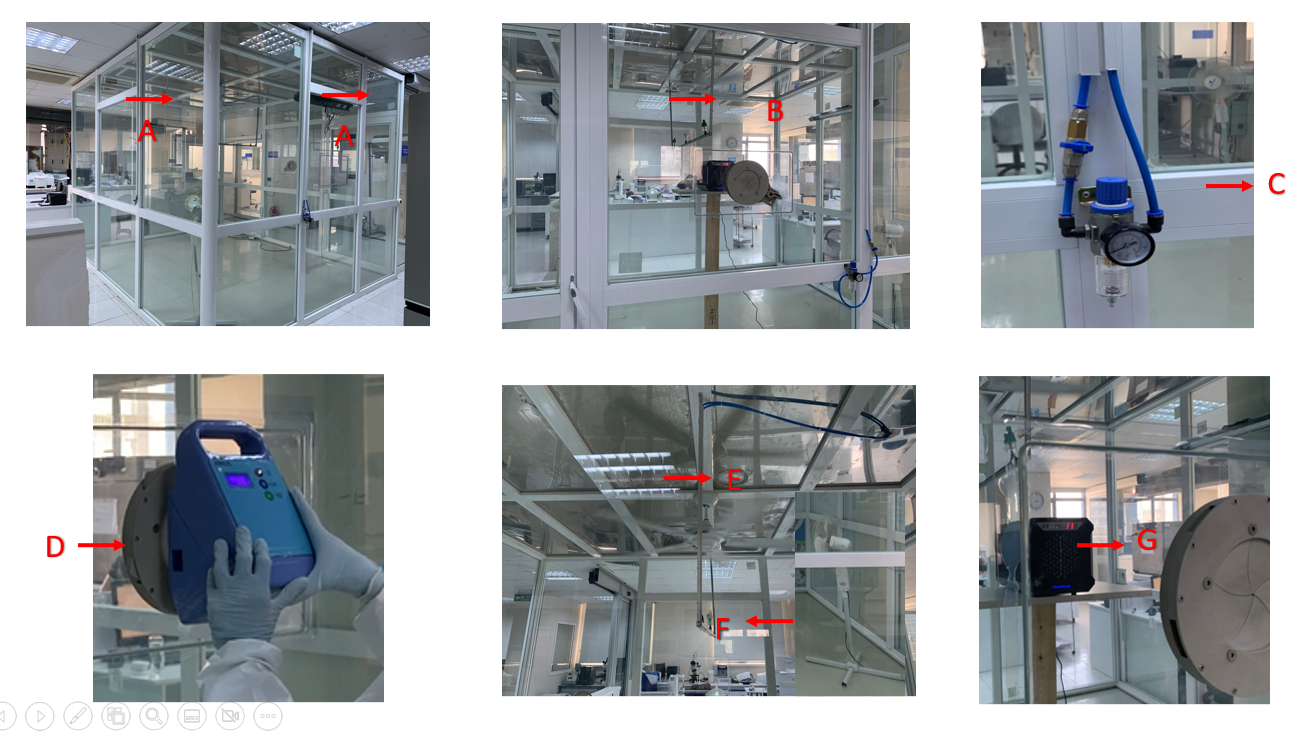
**

**Figure S2**: 30m^3^ aerosol chamber for bacterial test

**A:** UVC **B:** Nebulizer **C:** Bacteria flow **D:** Sampler  **E:** Ceiling Fan **F :** Stand fan **G:** NPBI device


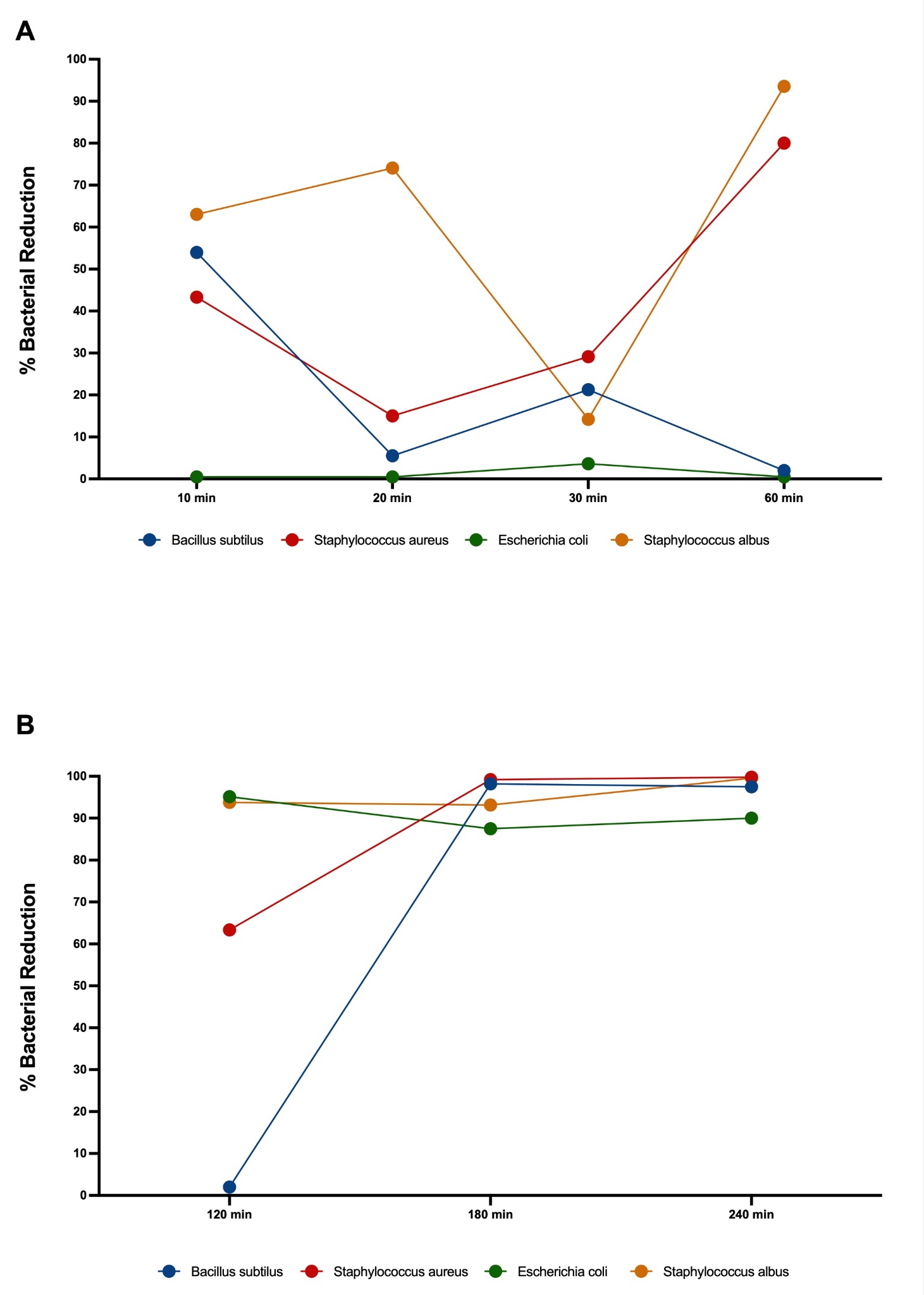


**Figure S3.** Bacterial reduction rate within 1 hour (A) and between 2 and 4 hours (B) during the operation of NPBI device in comparison with natural decay.


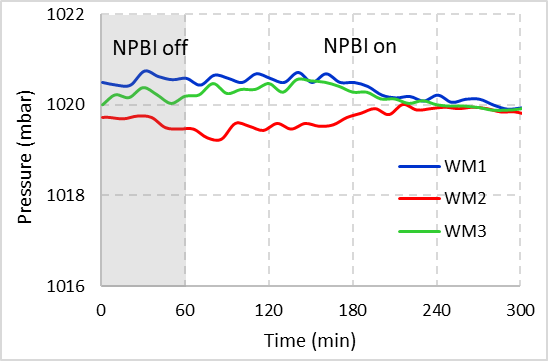

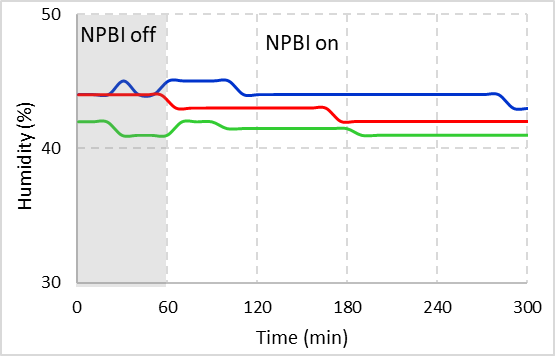

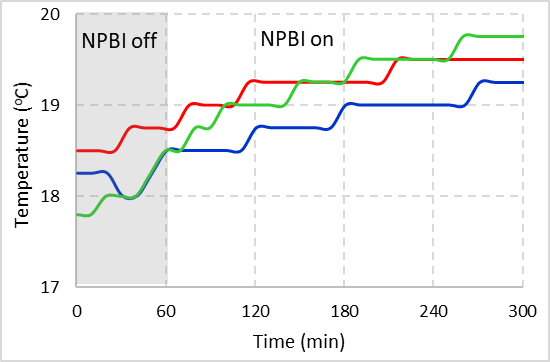

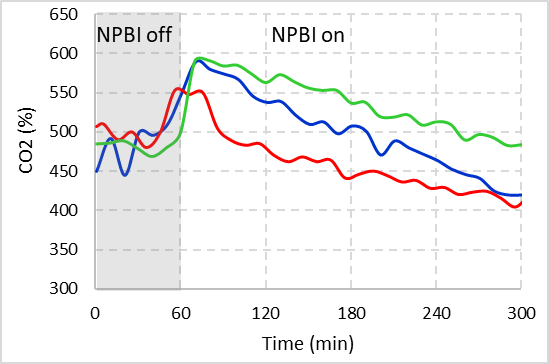


**Figure S4.** Thermal comfort parameters and CO_2_ in the office room before and during the operation of NPBI device WM_1_: 2.68 m^3^/min; WM_2_: 3.26 m^3^/min; WM_3_: 3.88 m^3^/min.


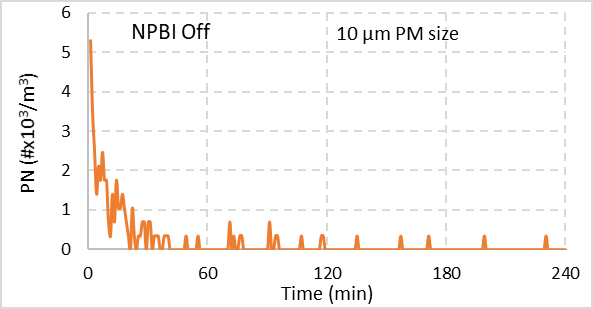

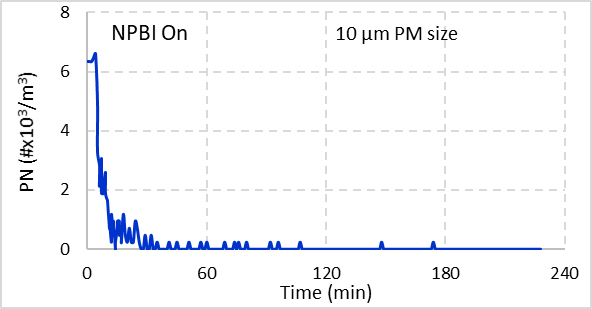


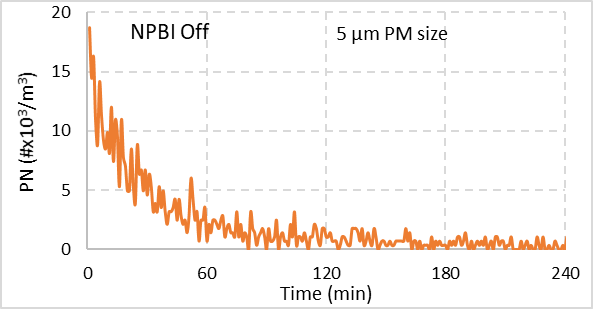

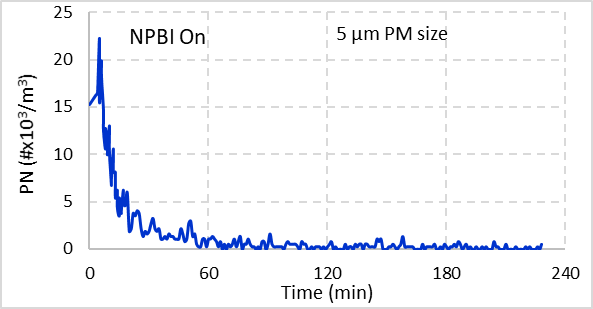

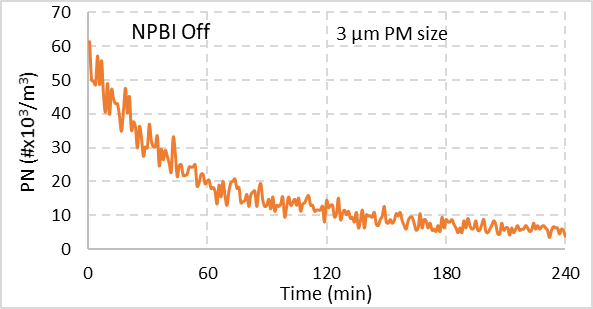

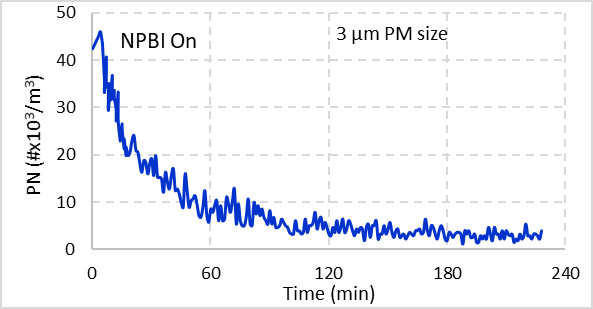

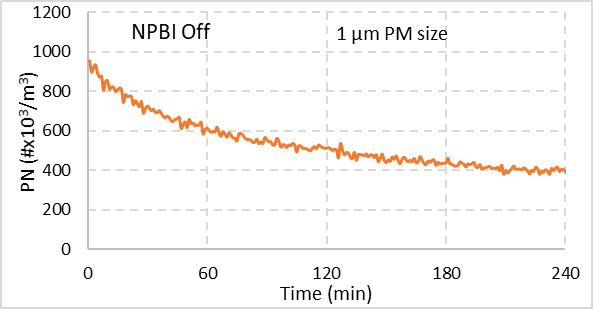

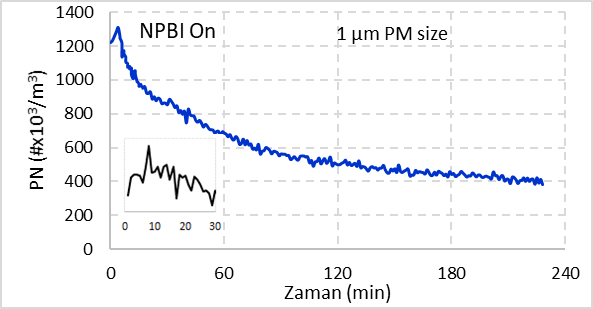

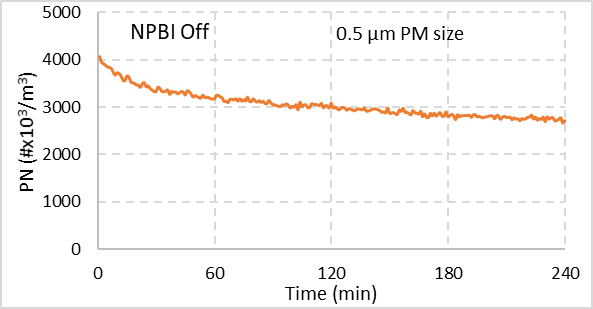

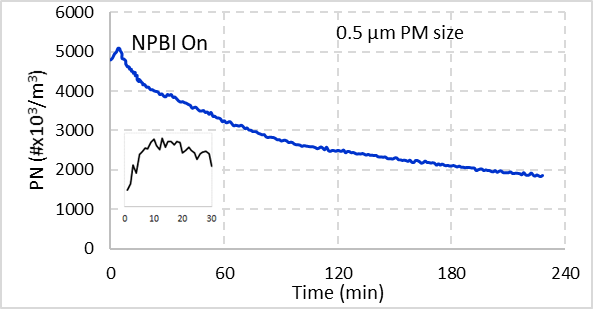

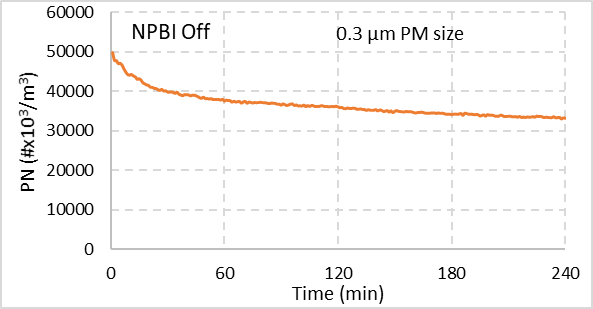

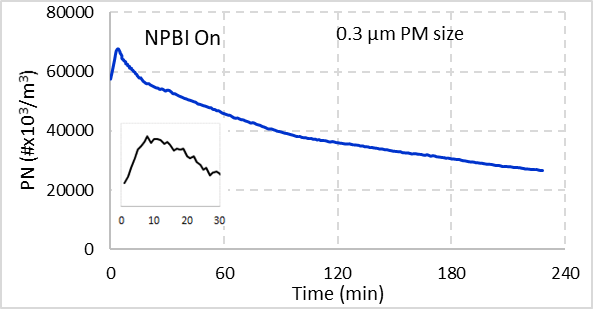


**Figure S5.** Particle Number Count Change during the NPBI device on (left) and off (right) in the office room. Small graphs for 0.3, 0.5 and 1 um PM sizes on the left side graphs are focusing on the first 30 minutes when NPBI device on.

**Table S1**: The microbial test results.

| *Bacteria* | Time (minute) | Log10 Mean Natural (NPBI off) | Log10 Mean  (NPBI on) |
| --- | --- | --- | --- |
| *Bacillus subtilis* | 0 | 6.457 | 5.358 |
|  | 10 | 6.164 | 4.829 |
|  | 20 | 5.735 | 4.709 |
|  | 30 | 5.674 | 4.568 |
|  | 60 | 5.048 | 4.049 |
|  | 120 | 4.496 | 3.811 |
|  | 180 | 4.044 | 1.301 |
|  | 240 | 3.329 | 1.000 |
| *Staphylococcus aureus* | 0 | 7.769 | 5.477 |
|  | 10 | 7.403 | 4.618 |
|  | 20 | 7.135 | 4.584 |
|  | 30 | 6.847 | 3.712 |
|  | 60 | 6.719 | 4.151 |
|  | 120 | 6.080 | 3.248 |
|  | 180 | 5.740 | 2.954 |
|  | 240 | 5.348 | 0.523 |
| *Escherichia coli* | 0 | 6.295 | 5.652 |
|  | 10 | 5.827 | 4.875 |
|  | 20 | 5.619 | 4.799 |
|  | 30 | 5.530 | 4.505 |
|  | 60 | 4.767 | 4.397 |
|  | 120 | 4.169 | 1.845 |
|  | 180 | 3.527 | 1.602 |
|  | 240 | 3.322 | 1.301 |
| *Staphylococcus albus* | 0 | 6.710 | 5.602 |
|  | 10 | 6.635 | 5.195 |
|  | 20 | 6.622 | 5.019 |
|  | 30 | 5.819 | 4.741 |
|  | 60 | 5.718 | 3.518 |
|  | 120 | 5.224 | 2.982 |
|  | 180 | 5.153 | 2.892 |
|  | 240 | 4.857 | 1.000 |

**Table S2:** The p values of t-test between the average values of the device-off (during a hour) and for each one hour time spans during the device-on.

| Fan Mode of the device | Minutes after the device work on | Parameters |  |  |  |  |  |  |
| --- | --- | --- | --- | --- | --- | --- | --- | --- |
|  |  | Pressure | Temperature | Humidity | CO2 | VOC | PM2.5 | NO2 |
| WM1 | 60-120 | 0.65 | 0.00 | 0.05 | 0.00 | 0.00 | 0.00 | 0.17 |
|  | 120-180 | 0.48 | 0.00 | 0.92 | 0.00 | 0.00 | 0.01 | 0.09 |
|  | 180-240 | 0.01 | 0.00 | 0.30 | 0.61 | 0.00 | 0.05 | 0.00 |
|  | 240-300 | 0.00 | 0.00 | 0.10 | 0.01 | 0.00 | 0.00 | 0.00 |
| WM2 | 60-120 | 0.00 | 0.00 | 0.36 | 0.15 | 0.00 | 0.35 | 0.01 |
|  | 120-180 | 0.03 | 0.00 | 0.08 | 0.00 | 0.00 | 0.01 | 0.18 |
|  | 180-240 | 0.00 | 0.00 | 0.00 | 0.00 | 0.19 | 0.00 | 0.50 |
|  | 240-300 | 0.00 | 0.00 | 0.00 | 0.00 | 0.00 | 0.00 | 0.05 |
| WM3 | 60-120 | 0.02 | 0.00 | 0.23 | 0.00 | 0.00 | 0.00 | 0.00 |
|  | 120-180 | 0.00 | 0.00 | 0.73 | 0.00 | 0.00 | 0.23 | 0.00 |
|  | 180-240 | 0.57 | 0.00 | 0.12 | 0.00 | 0.00 | 0.01 | 0.03 |
|  | 240-300 | 0.00 | 0.00 | 0.06 | 0.02 | 0.00 | 0.00 | 0.31 |
